# Diagnostic and Therapeutic Challenges of Cerebral Venous Thrombosis in SARS-CoV-2 Infection: A Case Report and Review of Literature

**DOI:** 10.3390/clinpract11030075

**Published:** 2021-09-07

**Authors:** Faisal Khan, Neha Sharma, Moin Ud Din, Ryan Chetram

**Affiliations:** 1 Neurology, College of Osteopathic Medicine, Sam Houston State University, Huntsville, TX 77340, USA; 2 Neurology, Houston Medical Clerkship, Sugar Land, TX 77478, USA; NehaSharmaMD@hotmail.com (N.S.); moinuddinbaber@gmail.com (M.U.D.); 3 School of Medicine, Caribbean Medical University, 4797 Willemstad, Curaçao; ryanchetram@hotmail.com

**Keywords:** COVID-19, severe acute respiratory syndrome coronavirus, anticoagulants, case report, cerebral venous thrombosis, brain ischemia

## Abstract

Headache, a common prodromal symptom of the severe acute respiratory syndrome coronavirus 2 (SARS-CoV-2) infection, can also be a manifestation of cerebral venous thrombosis (CVT), secondary to COVID-19. CVT management continues to evolve, with direct oral anticoagulants (DOACs) emerging as an alternative to warfarin. A 44-year-old Asian female, with no past medical history, presented to the emergency room (ER) with complaints of nonproductive cough and left-sided headache. She denied a history of COVID-19 vaccination, and SARS-CoV-2 testing (with reverse transcriptase-polymerase chain reaction) was positive. Non-contrast computed tomography (CT) of the head revealed left transverse sinus hyperdensity, consistent with dense vein sign, and magnetic resonance venography (MRV) confirmed the presence of thrombus. The initial treatment included subcutaneous enoxaparin with headache resolution, and she was discharged on apixaban. Five weeks later, a non-contrast head CT showed resolution of the dense vein sign and recanalisation of left transverse sinus was seen on MRV. This report has highlighted the need for increased awareness of coagulopathy and thrombotic events, including cerebral venous thrombosis, in patients infected with SARS-CoV-2. Unremitting headache, in context of SARS-CoV-2 infection, should be evaluated with appropriate neurovascular imaging. Controlled studies are required to compare the safety and efficacy of DOACs with warfarin for management of cerebral venous thrombosis.

## 1. Introduction

Headache is one of the most commonly reported initial manifestations of severe acute respiratory syndrome coronavirus 2 (SARS-CoV-2) infection (50%) [1]. This has been suggested to be secondary to systemic inflammatory response syndrome. Rarely, these headaches can also signify the presence of an underlying CVT, secondary to the thrombogenic state induced by SARS-CoV-2. Isolated headaches can be the sole initial manifestation of CVT, irrespective of underlying etiology, in 14% of cases [2,3]. Development of CVT, secondary to SARS-CoV-2 infection, is being increasingly recognized.

The COVID-19 pandemic has made the use of warfarin challenging, as it requires prolonged hospital stays and outpatient monitoring to establish optimum International Normalized Ratio (INR) [4,5]. Direct oral anticoagulants (DOACs) have been increasingly used for prophylaxis of strokes and systemic embolism, and have also emerged as a viable treatment option for CVT [6,7]. These anticoagulants have shown a comparable efficacy and an improved safety profile, as compared to warfarin, for the management of CVT [6,7]. The pharmacodynamic properties of DOACs provide an added advantage (by providing immediate therapeutic effect) and require no additional monitoring [5]. Therefore, there has been an increase in the use of DOACS for CVT during the COVID-19 pandemic.

Here, we present a case of a young female who presented with an isolated headache and was found to have left transverse sinus thrombosis, secondary to SARS-CoV-2 infection. We also performed a literature search, focusing on cases that used DOACs for the management of CVT, in the context of SARS-CoV-2 infection (Table 1) [1,8,9,10,11,12,13,14].

## 2. Case Report

A 44-year-old Vietnamese female, with no significant past medical history, presented to the emergency room (ER) with a three-week history of nonproductive cough and left-sided, pressure-like headache of moderate intensity (6/10). The patient denied fevers, chills, sore throat, and exposure to sick contacts with SARS-CoV-2 infection. The patient denied COVID-19 vaccination. She also denied dizziness, neck stiffness, and changes to her mental status. The headache and cough were refractory to the azithromycin (Z-pak) and steroids prescribed by her primary care physician one week prior. The patient denied a personal history of smoking, alcohol, illicit drug, and oral contraceptive use. She reported family history of diabetes mellitus, but denied hypertension, hyperlipidemia, and hypercoagulable disorders. Vital signs taken on arrival were blood pressure 104/57 mmHg, heart rate 102 bpm, respiratory rate 20 breath/min, temperature 98.4 °F, SpO_2_ of 99% on room air, and weight of 60 kg. Physical examination revealed the patient was alert and oriented to person, place, time, and situation. Cardiopulmonary examination was unremarkable. Neurological examination showed no focal motor or sensory deficits, intact cranial nerves I-XII, and no signs of meningeal irritation (negative Kernig and Brudzinski signs).

**Figure 1:**
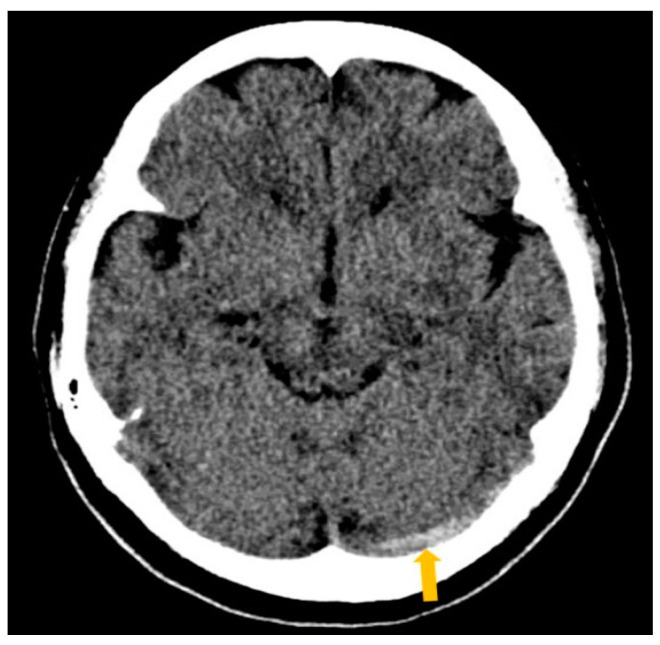
Non-contrast brain computed tomography (CT) at first emergency room visit revealed an asymmetric hyperdensity of the left transverse sinus (dense vein sign), consistent with acute thrombosis with no associated parenchymal edema or hemorrhage.

Intravenous fluids, acetaminophen, and ketorolac were administered, providing minimal pain relief. The complete blood count showed hemoglobin (Hb) 13.4 g/dL, mean corpuscular volume (MCV) 87.6 g/L, white blood cell count (WBC) 7.8 × 10^9^/L, lymphocytes 29.2%, monocytes 12.7%, and platelets 296 × 10^9^/L. The comprehensive metabolic panel was within normal limits. Hologic’s Panther Fusion^®^ SARS-CoV-2 testing, with a transcription-mediated polymerase chain reaction (performed in Houston, TX, USA) was positive. Chest X-ray showed the lungs were clear, with no evidence of consolidation, masses, pleural effusion, or pneumothorax. The pulmonary vasculature, cardiac, and mediastinal contours were also within normal limits. Despite pain management, the patient continued to complain of left-sided headache, prompting neuroimaging. Non-contrast brain CT revealed an asymmetric hyperdensity of the left transverse sinus, consistent with acute thrombosis with no associated parenchymal edema or hemorrhage (Figure 1).

**Figure 2:**
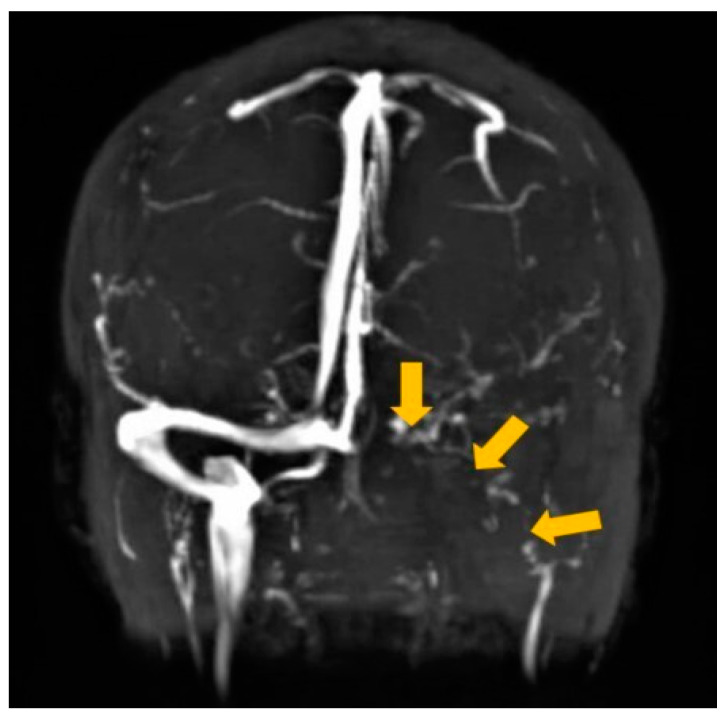
Magnetic resonance venography (MRV) on admission revealed a lack of flow-related signal in the left transverse sinus, correlating with dense vein sign seen on non-contrast head computed tomography (CT), confirming the presence of cerebral venous thrombosis. Additionally, there was evidence of hypoplasia in the sigmoid sinus and jugular bulb.

The patient was admitted and placed in negative-pressure isolation. Therapeutic anticoagulation, with 60 mg/0.6 mL subcutaneous enoxaparin twice per day (BID), was initiated. Non-contrast brain magnetic resonance imaging (MRI) showed no infarcts or hemorrhages. Magnetic resonance venography (MRV) revealed lack of flow-related signal in the left transverse sinus, correlating with dense vein sign seen on the non-contrast head CT, confirming the presence of cerebral venous thrombosis. Additionally, there was evidence of hypoplasia in the left sigmoid sinus and jugular bulb (Figure 2). Additional laboratory testing revealed homocysteine 6.0 umol/L and negative titers for antinuclear antibody, anticardiolipin antibody, beta-2 glycoprotein antibody, and lupus anticoagulant. Coagulation panel was negative for Factor G20210 A and Factor V Leiden mutations, with an Antithrombin III of 126%, a PTT 28.9 s, PT 13 s, INR 0.98, Protein C 112 IU, and Protein S 54 IU. On day three, the patient was transitioned onto 10 mg apixaban BID. The patient’s headache resolved on the fifth day of admission. She was discharged home, initially on apixaban 10 mg BID for four more days to complete the seven-day course, and then was switched to apixaban 5 mg BID, with additional instructions to follow up outpatient for a repeat MRV.

**Figure 3:**
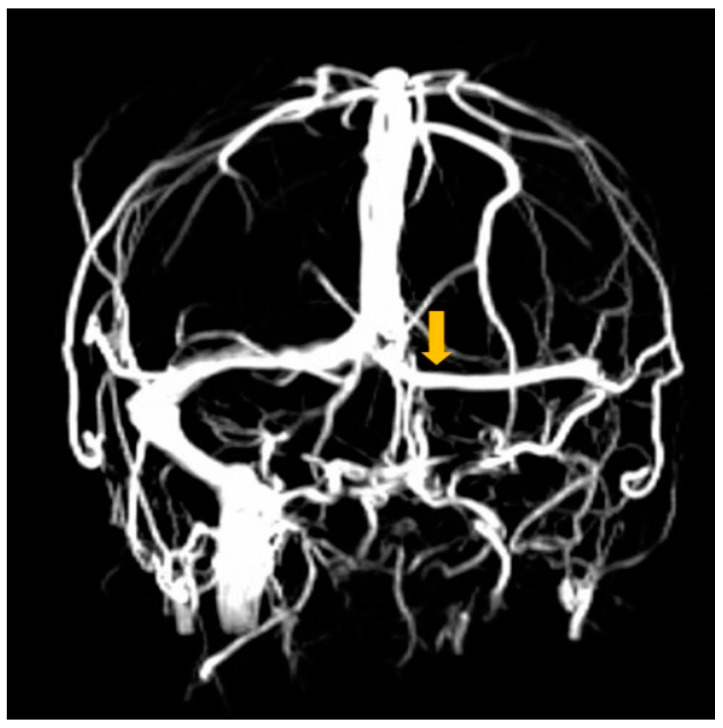
Magnetic resonance venography (MRV), five weeks later, revealed recanalization of the small caliber left transverse sinus with persistent hypoplasia of sigmoid sinus and jugular bulb.

Five weeks later, the patient returned to the ER, due to a one-week history of intermittent holocranial pressure, refractory to acetaminophen. Vitals revealed blood pressure 106/77 mmHg, heart rate 109 bpm, respiratory rate 18 breaths/min, temperature 98.1 °F, and SpO_2_ of 97% on room air. Physical examination revealed that the patient was alert and oriented to person, place, time, and situation. Cardiopulmonary examination was unremarkable. Neurological examination showed no cranial nerve deficits, intact speech, no focal motor or sensory deficits, and no signs of meningeal irritation (negative Kernig and Brudzinski signs). Complete blood count and comprehensive metabolic panel were normal. Coagulation screening showed INR 1.05. She reported compliance with the apixaban 5 mg, twice daily. Non-contrast head CT did not show any acute intracranial abnormalities with resolution of the left transverse sinus dense vein sign. Recanalization of the small caliber left transverse sinus with persistent hypoplasia of sigmoid sinus and jugular bulb was seen on MRV (Figure 3). The patient was offered admission for symptomatic management of headaches but left against medical advice and was lost to follow up.

## 3. Discussion

Headaches are being frequently reported as a prodromal symptom of SARS-CoV-2 infection. Caronna et al. reported that 21.4% of patients had a headache as their first clinical manifestation of SARS-CoV-2 infection and hypothesized that it could be caused by systemic inflammatory response processes [15]. These headaches were new-onset (24.7%), moderate intensity (50.6%), and characterized as pressing (70.1%) or throbbing (19.6%) [15]. New-onset or intractable headaches, associated with the SARS-CoV-2 infection, can also be the sole manifestation of CVT (as seen in our patient) and requires thorough evaluation. Isolated headache (14%), is the most common presenting symptom of both provoked and unprovoked CVT [2,3]. Headaches, secondary to CVT with an underlying SARS-CoV-2 infection, affect approximately 50% of patients [1]. These headaches are commonly unremitting and can be associated with loss of consciousness (30.8%) and seizures (19.5%) [1,11]. 

**Scheme 1:**
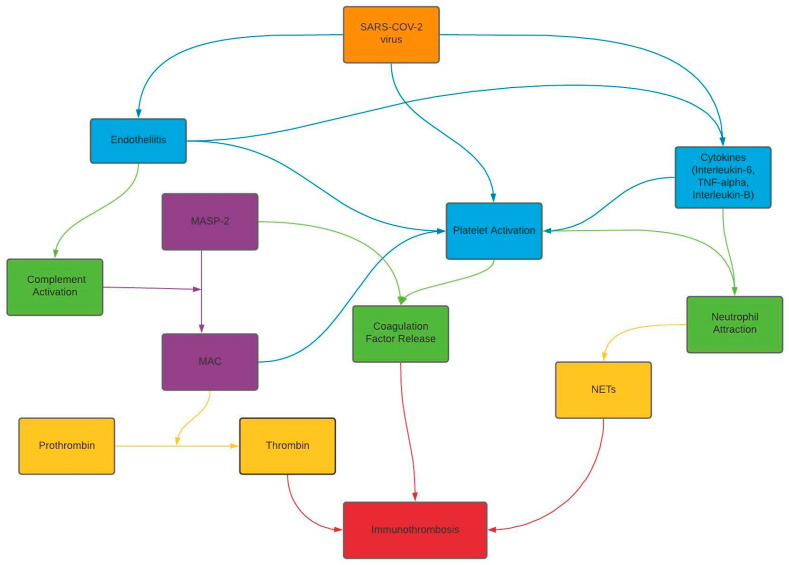
Flow chart depicting the multiple factors contributing to the hypothesized mechanisms of thrombus formation secondary to SARS-CoV-2 virus infection.

Cerebrovascular complications, secondary to SARS-CoV-2 infection, are becoming more frequently reported [11,16]. Cerebral venous thrombosis is now a well-established complication in SARS-CoV-2 infection, with a reported incidence of 4.5/100,000 [11]. Chaumont et al. published the first case of COVID-19 and CVT in May 2020 [16]. The SARS-CoV-2 virus has been hypothesized to contribute to thrombus formation through a multifactorial pathway (Scheme 1). The virus is hypothesized to indirectly damage the endothelial cells secondary to a cascade of inflammatory events ultimately leading to the formation of immunothrombus: a microthrombus composed of citrullinated histone H3 positive neutrophils, platelets, and fibrin [17,18,19].

Diagnosing CVT requires the utilization of various neurovascular imaging modalities. The dense vein sign and dense triangle/clot signs on non-contrast head CT have a low sensitivity though high specificity [20,21]. Computed tomography venography (CTV) and MRV have high sensitivity and specificity for diagnosing CVT, and (in rare cases) cerebral angiography is required if findings are inconclusive [22,23]. Our patient presented with an isolated headache, and the dense vein sign was seen on non-contrast CT (Figure 1), correlating with the absence of flow-related signal on MRV (Figure 2), confirming the presence of CVT. Additionally, the SARS-CoV-2 reverse transcriptase-polymerase chain reaction test (100% specificity) [24] confirmed COVID-19 infection. Baldini et al. reported that in SARS-CoV-2 infected patients with CVT, 31% had concurrent, underlying conditions contributing to thrombus formation [25]. Although the SARS-CoV-2 infection has been linked to thrombogenic states, it is imperative for providers to evaluate the underlying diseases that contribute to a hypercoagulable state. Appropriate history and diagnostic testing ruled out secondary etiologies of the CVT. These diagnostic findings led to the conclusion that, in this patient, the underlying etiology of CVT was SARS-CoV-2 infection.

Cerebral venous thrombosis, in the context of SARS-CoV-2 infection, was first reported by Chaumont et al. in May 2020 [16]. In order to evaluate the use of DOACs in CVT and the COVID-19 disease, we performed a literature search (using PubMed and Google Scholar) for English language articles from 2020 to 2021, using the key terms “cerebral venous thrombosis”, “direct oral anticoagulant”, and “COVID-19”. The main exclusion criteria used were (1) lack of cerebral venous thrombosis, (2) cerebral venous thrombosis without COVID-19, and (3) lack of DOAC use. Seven publications, as of 16 July 2021, were found depicting the use of DOACs in patients with CVT and COVID-19 (Table 1) [8,9,10,11,12,13,14].

Therapeutic doses of low molecular weight heparin should be initiated for the acute treatment of CVT, even in cases when venous strokes are complicated by hemorrhagic conversion [4]. The use of anticoagulation has been proven to be safe and effective, as it is directed towards the underlying pathology of thrombosis. In patients with cancers associated with acute thrombosis, the use of DOACs is preferred over low molecular weight heparin (LMWH), with the exception of gastrointestinal or urological cancers [26]. The gold-standard, long-term treatment of CVT is warfarin, with a target INR of 2.0–3.0 [4,6]. Warfarin has been reported to be the most effective anticoagulation therapy, compared to DOACs, in patients with antiphospholipid syndrome, prosthetic valves, and those with high risk of gastrointestinal bleeds [26,27]. For warfarin to be therapeutic, prolonged stays in the hospital are required, with additional long-term outpatient INR monitoring. The COVID-19 pandemic has made management of the INR difficult. The pandemic has caused an overcrowding of hospitals, thus increasing the risk of exposure to the SARS-CoV-2 virus. Furthermore, it has increased cost and decreased availability of in-home INR monitoring kits. Our patient had additional challenges, such as communication issues (Vietnamese speaking only) and lack of transportation. Taking all of these factors into consideration, we chose to start the patient on apixaban 5 mg, which proved to be effective, as our patient had resolution of the cerebral venous thrombosis and was safe with no neurological complications.

A few limitations in our study include missing D-dimer and fibrinogen levels. D-Dimer is helpful in diagnosing thromboembolic conditions, especially deep venous thrombosis and pulmonary embolisms. In this case, CVT was seen on the initial CT scan in the ER, which led to further neurovascular imaging, confirming the diagnosis. Another oversight, on our part, was not obtaining JAK2 V617 F mutation levels. However, there was low suspicion for underlying myeloproliferative disorders causing thrombosis, due to the patient lacking any corresponding history, clinical features, and laboratory findings. Although, there has been one rare reported case of the development of CVT as the initial presentation of myeloproliferative disorders [28].

The overall mortality of patients with SARS-CoV-2 and CVT has been reported to be 35–45%, as compared to CVT alone (3.3–15%), or COVID-19 disease alone (5.6%) [11,29]. The proposed pathogenesis of higher mortality includes increased thrombus burden and more frequent involvement of the deep venous sinuses (straight sinus and internal cerebral veins) [11,29]. However, larger studies are needed to determine the overall incidence and mortality related to CVT in SARS-CoV-2 infected patients.

## 4. Conclusions

The COVID-19 disease spectrum and its related systemic and neurological complications continue to evolve and present new management challenges. Headache is a frequently reported initial manifestation of the SARS-CoV-2 infection. Irrespective of the underlying etiology, headache can also be the sole clinical presentation of underlying CVT. This report has highlighted the need for an increased awareness of coagulopathy and thrombotic events, including cerebral venous thrombosis, in patients infected with SARS-CoV-2. In our patient, apixaban demonstrated appropriate efficacy and safety, which was in accordance with our literature review findings. However, further controlled studies are required to compare the overall efficacy and safety of DOACs with warfarin, for the long-term management of cerebral venous thrombosis.

## Figures and Tables

**Table 1:** Review of literature: cases involving cerebral venous thrombosis with COVID-19 that only used Direct Oral Anticoagulants for treatment.

Article	Study	Clinical Presentation	Location	Diagnostic Imaging	COVID-19 Diagnosis	Therapeutics	Outcome
Hughes 2020 [8]	Case Report	Right-sided frontotemporal progressive headache, ipsilateral numbness, slurred speech, and expressive dysphasia	Right sigmoid and transverse sinus	Head CT ^a^: hyperdensity of superior sagittal sinus, right transverse sinus, sigmoid sinus, and right upper jugular vein. CTV ^b^: filling defect in right sigmoid and transverse sinus involving the torcula	Nasopharyngeal COVID-19 ^c^ swab	1. Low molecular weight Heparin for 24 h (dosage unknown) 2. Apixaban 10 mg ^d^ BID ^e^ for 7 days	Patient is at home recovering as of publication.
Sugiyama 2020 [9]	Case Report	Fever and malaise	Confluence of sinus to left transverse sinus	Non-contrast CT ^a^ Head: hyperdensity of left transverse sinus (cord sign) T2-weighted MRI ^f^ demonstrated isointensity, T2-FLAIR ^g^ MRI ^f^ hyperintensity in the left transverse sinus T2-weighted MRI ^f^ hypointensity left transverse sinus.	First SARS-CoV-2 ^h^ RT PCR ^i^ test was negative. Repeat SARS-CoV-2 ^h^ RT PCR ^i^ was positive.	1. subcutaneous unfractionated Heparin 10,000 units (duration unknown); switched to IV ^m^ unfractionated Heparin, dose adjusted (specific dose unknown) for 18 days 2. Edoxaban 60 mg ^d^ QD ^j^ duration of at least 29 days	Significant improvement of sinus thrombus and discharged on day 33; continued Edoxaban and remained free of symptoms for 14 days after discharge.
Thompson 2020 [10]	Case Report	Delirium, executive dysfunction and dyspraxia	Superior sagittal sinus, left transverse sinus and left sigmoid sinus down to the level of the jugular foramen	Non-contrast CT ^a^ of the head was normal 1 week later: Repeat Head CT ^a^ and CTV ^b^: thrombosis of superior sagittal sinus, left transverse sinus, left sigmoid sinus, jugular foramen, and the vein of Labbé 7 × 8 mm ^l^ parenchymal haemorrhage in left temporal lobe 2 weeks from admission: radiological improvement with recanalisation of the vein of Labbe, partial recanalisation of the left transverse sinus and superior sagittal sinus. Acute haemorrhage within the slender hygroma.	Negative PCR ^k^ nasopharyngeal swabs for SARS-CoV-2 ^h^ (however probable COVID-19 ^c^ diagnosis was made, based on European Centre for Disease Control case definition)	1. subcutaneous Enoxaparin 40 mg ^d^ QD ^j^ for 7 days, switched to IV ^m^ Heparin 1.5 mg/kg QD ^j^ (duration unknown) 2. Apixaban 5 mg ^d^ BID ^e^ for minimum 3–6 months (personal communication)	Discharged from hospital and positive response to treatment to date of publication. Continuing anticoagulation for a minimum of 3-6 months.
Tu 2020 [11]	Case series Systematic Review	1. Chest pain, fever, and chills	1. Left transverse, sigmoid sinus	Not Applicable	Nasopharyngeal swab SARS-CoV-2 ^h^ RT PCR^i^ was positive.	1.Dabigatran (dosage/duration unknown)	1. Resolution of Cerebral Venous Thrombosis after 4 weeks
Bolaji 2020 [12]	Case report	Left sided weakness, left facial twitch, inability to stand, and left-sided extensor plantar response	Right transverse sinus	CT ^a^, CTV ^b^ revealed venous sinus thrombosis, bilateral venous cortical infarcts, acute cortical hemorrhage	Nasopharyngeal swab SARS-CoV-2 ^h^ positive	1. Therapeutic doses of IV ^m^ low molecular weight Heparin (specific dose unknown, started on admission, full duration unknown) 2. Edoxaban (dosage/duration unknown)	Discharged to long-term care facility for physical therapy and monitoring, then discharged home
Pang 2021 [13]	Case report	3 day history of fever, cough, and headache	Left transverse and sigmoid sinuses	MRI ^f^ Brain, MRV ^n^ (T2 flow void)	RT-PCR ^i^ Nasopharyngeal swab SARS-CoV-2 ^h^ positive	1. Heparin use unknown 2. Dabigatran 150 mg ^d^ BID ^e^ for 3 months	CTV ^b^ 1 month later revealed resolution, then patient was lost to follow up
Hameed 2021 [14]	Multicenter Study and Review of Literature	Headache, seizures, altered mental status, hemiparesis, and heminumbness	Superior sagittal, transverse, sigmoid, cavernous, straight sinuses and internal jugular vein	Neuroimaging revealed infarction in affected sinuses	RT-PCR ^i^ Nasopharyngeal swab SARS-CoV-2 ^h^ positive	1.Low molecular weight Heparin (dosage/duration unknown) 10 of 18 patients 2.Unfractionated Heparin (dosage/duration unknown) 4 of 18 patients 3. Direct oral anticoagulants (dosage/duration unknown) 6 of 18 patients; Rivaroxaban (dosage/duration unknown) 5 of 6 patients; Dabigatran (dosage/duration unknown) 1 of 6 patients	mRS ^o^ score 0–2 ^o^

Abbreviations: a—computed tomography (CT); b—computed tomography venography (CTV); c—coronavirus disease 2019 (COVID-19); d—milligrams (mg); e—twice daily (BID); f—magnetic resonance imaging (MRI); g—fluid-attenuated inversion recovery (FLAIR); h—severe acute respiratory syndrome coronavirus 2 (SARS-CoV-2); i—reverse transcriptase-polymerase chain reaction (RT-PCR); j—once daily (QD); k—polymerase chain reaction (PCR); l—millimeters (mm); m—intravenous (IV); n—magnetic resonance venography (MRV); o—modified Rankin Scale (mRS).

## Data Availability

Not applicable.

## References

[1] Abdalkader M., Shaikh S.P., Siegler J.E., Cervantes-Arslanian A.M., Tiu C., Radu R.A., Tiu V.E., Jillella D.V., Mansour O.Y., Vera V. (2021). Cerebral Venous Sinus Thrombosis in COVID-19 Patients: A Multicenter Study and Review of Literature. J. Stroke Cerebrovasc. Dis..

[2] Alons I.M., Jellema K., Wermer M.J., Algra A. (2015). D-dimer for the exclusion of cerebral venous thrombosis: A meta-analysis of low risk patients with isolated headache. BMC Neurol..

[3] Khan F., Seyam M., Sharma N., Din M.U., Bansal V. (2021). New Horizons for Diagnostic Pitfalls of Cerebral Venous Thrombosis: Clinical Utility of a Newly Developed Cerebral Venous Thrombosis Diagnostic Score: A Case Report and Literature Review. Am. J. Case Rep..

[4] Ferro J.M., Bousser M., Canhão P., Coutinho J.M., Crassard I., Dentali F., di Minno M., Maino A., Martinelli I., Masuhr F. (2017). European Stroke Organization guideline for the diagnosis and treatment of cerebral venous thrombosis—Endorsed by the European Academy of Neurology. Eur. J. Neurol..

[5] Chen A., Stecker E., Warden B.A. (2020). Direct Oral Anticoagulant Use: A Practical Guide to Common Clinical Challenges. J. Am. Heart Assoc..

[6] Ferro J.M., Coutinho J.M., Dentali F., Kobayashi A., Alasheev A., Canhão P., Karpov D., Nagel S., Posthuma L., Roriz J.M. (2019). Safety and Efficacy of Dabigatran Etexilate vs. Dose-Adjusted Warfarin in Patients With Cerebral Venous Thrombosis. JAMA Neurol..

[7] Hsu A., Mistry H., Lala N., Reagan J.L. (2020). Preliminary findings regarding the use of direct oral anticoagulants in cerebral venous thrombosis. Clin. Neurol. Neurosurg..

[8] Hughes C., Nichols T., Pike M., Subbe C., Elghenzai S. (2020). Cerebral Venous Sinus Thrombosis as a Presentation of COVID-19. Eur. J. Case Rep. Intern. Med..

[9] Sugiyama Y., Tsuchiya T., Tanaka R., Ouchi A., Motoyama A., Takamoto T., Hara N., Yanagawa Y. (2020). Cerebral venous thrombosis in COVID-19-associated coagulopathy: A case report. J. Clin. Neurosci..

[10] Thompson A., Morgan C., Smith P. (2020). Cerebral venous sinus thrombosis associated with COVID-19. Am. J. Neuroradiol..

[11] Tu T.M., Goh C., Tan Y.K., Leow A.S., Pang Y.Z., Chien J., Shafi H., Chan B.P., Hui A., Koh J. (2020). Cerebral Venous Thrombosis in Patients with COVID-19 Infection: A Case Series and Systematic Review. J. Stroke Cerebrovasc. Dis..

[12] Bolaji P., Kukoyi B., Ahmad N., Wharton C. (2020). Extensive cerebral venous sinus thrombosis: A potential complication in a patient with COVID-19 disease. BMJ Case Rep..

[13] Pang Y.Z., Shafi H., Lee Z.C., Ting S.K.S., De Silva D.A. (2021). Cerebral venous thrombosis in a patient with mild COVID-19 infection. Ann. Acad. Med. Singap..

[14] Hameed S., Wasay M., Soomro B.A., Mansour O., Abd-Allah F., Tu T., Farhat R., Shahbaz N., Hashim H., Alamgir W. (2021). Cerebral Venous Thrombosis Associated with COVID-19 Infection: An Observational, Multicenter Study. Cerebrovasc. Dis. Extra.

[15] Caronna E., Ballvé A., Llauradó A., Gallardo V.J., Ariton D.M., Lallana S., Maza S.L., Gadea M.O., Quibus L., Restrepo J.L. (2020). Headache: A striking prodromal and persistent symptom, predictive of COVID-19 clinical evolution. Cephalalgia.

[16] Chaumont H., Etienne P., Roze E., Couratier C., Roger P.-M., Lannuzel A. (2020). Acute meningoencephalitis in a patient with COVID-19. Rev. Neurol..

[17] McCracken I.R., Saginc G., He L., Huseynov A., Daniels A., Fletcher S., Peghaire C., Kalna V., Andaloussi-Mäe M., Muhl L. (2021). Lack of Evidence of Angiotensin-Converting Enzyme 2 Expression and Replicative Infection by SARS-CoV-2 in Human Endothelial Cells. Circulation.

[18] Varga Z., Flammer A.J., Steiger P., Haberecker M., Andermatt R., Zinkernagel A.S., Mehra M.R., Schuepbach R.A., Ruschitzka F., Moch H. (2020). Endothelial cell infection and endotheliitis in COVID-19. Lancet.

[19] Lou M., Yuan D., Liao S., Tong L., Li J. (2021). Potential mechanisms of cerebrovascular diseases in COVID-19 patients. J. NeuroVirol..

[20] Alvis H., Castellar-Leones S.M., Alcala-Cerra G., Moscote-Salazar L.R. (2013). Cerebral sinus venous thrombosis. J. Neurosci. Rural Pract..

[21] Tayyebi S., Akhavan R., Shams M., Salehi M., Farrokh D., Yousefi F., Abbasi B. (2020). Diagnostic value of non-contrast brain computed tomography in the evaluation of acute cerebral venous thrombosis. Sci. Rep..

[22] Dmytriw A.A., Song J.S.A., Yu E., Poon C.S. (2018). Cerebral venous thrombosis: State of the art diagnosis and management. Neuroradiology.

[23] Lee S.-K., Mokin M., Hetts S.W., Fifi J.T., Bousser M.-G., Fraser J.F. (2018). Current endovascular strategies for cerebral venous thrombosis: Report of the SNIS Standards and Guidelines Committee. J. Neurointerventional Surg..

[24] Sethuraman N., Jeremiah S.S., Ryo A. (2020). Interpreting Diagnostic Tests for SARS-CoV-2. JAMA.

[25] Baldini T., Asioli G.M., Romoli M., Dias M.C., Schulte E.C., Hauer L., De Sousa D.A., Sellner J., Zini A. (2021). Cerebral venous thrombosis and severe acute respiratory syndrome coronavirus-2 infection: A systematic review and meta-analysis. Eur. J. Neurol..

[26] Wojtukiewicz M.Z., Skalij P., Tokajuk P., Politynska B., Wojtukiewicz A.M., Tucker S.C., Honn K.V. (2020). Direct Oral Anticoagulants in Cancer Patients. Time for a Change in Paradigm. Cancers.

[27] Wadsworth D., Sullivan E., Jacky T., Sprague T., Feinman H., Kim J. (2021). A review of indications and comorbidities in which warfarin may be the preferred oral anticoagulant. J. Clin. Pharm. Ther..

[28] Sales C., Wijeratne T., Lucero A. (2021). Is JAK2-Mutation Associated with Extensive Clot Burden in Cerebral Venous Thrombosis?. Ann. Clin. Case Rep..

[29] Ostovan V.R., Foroughi R., Rostami M., Almasi-Dooghaee M., Esmaili M., Bidaki A.A., Behzadi Z., Farzadfard F., Marbooti H., Rahimi-Jaberi A. (2021). Cerebral venous sinus thrombosis associated with COVID-19: A case series and literature review. J. Neurol..

